# Oxidative and carbonyl stress induced AMD and *Codonopsis lanceolata* ameliorates AMD via controlling oxidative and carbonyl stress

**DOI:** 10.1038/s41598-024-67044-3

**Published:** 2024-07-15

**Authors:** Soon-Young Lee, Yeon-Kyoung Cho, Chun-Sik Bae, Gyeyeop Kim, Min-Jae Lee, Seung-Sik Cho, In-Chul Jeon, Dae-Hun Park

**Affiliations:** 1 College of Korean Medicine, Dongshin University, Naju, 58245 Jeonnam Korea; 2 College of Health and Welfare, Dongshin University, Naju, 58245 Jeonnam Korea; 3 College of Veterinary Medicine, Chonnam National University, Gwangju, 61186 Korea; 4 College of Veterinary Medicine, Kangwon National University, Chuncheon, 24341 Gangwon Korea; 5 Department of Biomedicine, Health & Life Convergence Sciences, BK21 Four, Biomedical and Healthcare Research Institute, Mokpo National University, Muan, 58554 Jeonnam Korea; 6 Department of Pharmacy, College of Pharmacy, Mokpo National University, Muan, 58579 Jeonnam Korea

**Keywords:** Age-related macular degeneration (AMD), *Codonopsis lanceolata*, Oxidative/carbonyl stress, Keap1/Nrf2/HO-1 pathway, 4-HNE, Apoptosis, Biological techniques, Drug discovery

## Abstract

Age-related macular degeneration (AMD) is one of the leading causes of blindness. AMD is currently incurable; the best solution is to prevent its occurrence. To develop drugs for AMD, it is crucial to have a model system that mimics the symptoms and mechanisms in patients. It is most important to develop safer and more effective anti-AMD drug. In this study, the dose of A2E and the intensity of blue light were evaluated to establish an appropriate atrophic in vitro model of AMD and anti-AMD effect and therapeutic mechanism of *Codonopsis lanceolata*. The experimental groups included a control group an AMD group treated with A2E and blue light, a lutein group treated with 25 μM lutein after AMD induction, and three groups treated with different doses of *C. lanceolata* (10, 20, and 50 μg/mL) after AMD induction. Intrinsic apoptotic pathway (Bcl-2 family), anti-oxidative system (Keap1/Nrf2/HO-1 antioxidant response element), and anti-carbonyl effect (4-hydroxynonenal [4-HNE]) were evaluated using immunofluorescence, MTT, TUNEL, FACS, and western blotting analyses. A2E accumulation in the cytoplasm of ARPE-19 cells depending on the dose of A2E. Cell viability of ARPE-19 cells according to the dose of A2E and/or blue light intensity. The population of apoptotic or necrotic cells increased based on the A2E dose and blue light intensity. *Codonopsis lanceolata* dose-dependently prevented cell death which was induced by A2E and blue light. The antiapoptotic effect of that was caused by activating Keap1/Nrf2/HO-1 pathway, suppressing 4-HNE, and modulating Bcl-2 family proteins like increase of antiapoptotic proteins such as Bcl-2 and Bcl-XL and decrease of proapoptotic protein such as Bim. Based on these findings, 30 μM A2E and 20 mW/cm^2^ blue light on adult retinal pigment epithelium-19 cells was an appropriate condition for AMD model and *C. lanceolata* shows promise as an anti-AMD agent.

## Introduction

Age-related macular degeneration (AMD) is a condition where the central vision is gradually lost as a result of aging. It is one of the leading causes of blindness worldwide^1^. In a meta-analysis conducted by the Wang group in 2022, the annual incidence of early and late AMD was found to be 1.59% and 0.23%, respectively. The study also projected that by 2050, the number of individuals affected by early and late AMD would reach 39.05 million and 6.41 million, respectively^2^. Another meta-analysis conducted in the United States revealed that the prevalence of AMD in individuals aged 40 years and older was 1.47%, while in women aged 80 years and older, it reached 15%^3^. This indicates a significant increase in the incidence of AMD with age. Furthermore, it is concerning that the population aged 60 years and older is projected to rapidly increase from 12% in 2015 to 22% in 2050^4^.

The bis-retinoid N-retinyl-N-retinylidene ethanolamine (A2E) is a type of lipofuscin that is fluorescent. During aging, it accumulates in various parts of the body, including neurons, cardiac muscle, and the retinal pigment epithelium (RPE)^5^. Exposure to blue light (415–455 nm) can stimulate damage to RPE cells through oxidative stress, ultimately leading to the development of AMD^6^. There are several risk factors that can contribute to the development of AMD such as aging, tobacco smoking, cataract surgery, family health history, and being overweight^7,8^. AMD can be classified into two types: atrophic AMD (85–90%), which is characterized by the presence of drusen and hyperpigmentation, and neovascular AMD (10–15%), which is characterized by the development of choroidal neovascularization following RPE cell death^9^.

The treatments for AMD are categorized into three types: device-based treatment (prophylactic laser therapy and photodynamic therapy), anti-inflammatory drug treatment (corticosteroids and nonsteroidal anti-inflammatory drugs), and anti-vascular endothelial growth factor (anti-VEGF) treatment (VEGF antibody, aptamer, and tyrosine kinase inhibitor)^10^. However, each approach has adverse effects. Device-based treatment can lead to macular edema and retinovascular disease^11,12^, while anti-inflammatory drug treatment can cause hypertension, insulin resistance, insomnia, skin thinning, and gastric ulceration^13^. Anti-VEGF treatment, specifically through intravitreal injection, carries the risk of bleeding and infection^14^.

Because of various adverse effects of AMD medication it is necessary to develop a safe and effective anti-AMD drug. In order to evaluate the anti-AMD effect and examine the therapeutic mechanisms of drug candidates, it is crucial to establish an appropriate model system. The cell-based AMD model can be classified into two types: A2E combined with blue light induction, and chemical induction. Several studies have explored the relationship between A2E dose and blue light intensity. For example, one study used 20 μM A2E and 0.04 mW/cm^2^ blue light for 48 h^15^, while others used 25 μM A2E and 4.43 mW/cm^2^ blue light for 30 min^16^, 100 μM A2E and 0.4 mW/cm^2^ blue light for 20 min, or 35 mW/cm^2^ blue light for 60 sec^17^. Many chemical stimulators that can induce AMD have been studied, including cigarette smoke^18^, H_2_O_2_^19^, KBrO_3_^20^, and NaIO_3_^21^. However, the advantages and disadvantages of each model are still unclear, and it is necessary to determine the differences among them.

*Codonopsis lanceolata* (*C. lanceolata*)has been used as a culinary ingredient and traditional medicinal material in Asia (Korea, China, and Japan) for a significant period of time. Recently, several biological effects of *C. lanceolata* have been reported, including its anti-proliferative effect on HT-29 colon cancer cells^22^, anti-obesity and anti-hyperlipidemia effects^23^, anti-hepatitis effect^24^, anti-inflammatory effect^25^, and anti-asthmatic effect^26^.

In this study, an appropriate in vitro model of AMD was suggested to effectively develop anti-AMD drugs. This model depended on the A2E and blue light treatment. Furthermore, the anti-AMD effect of *C. lanceolata*, along with its mechanism, was evaluated.

## Materials and methods

### A2E synthesis

**Figure 1:**
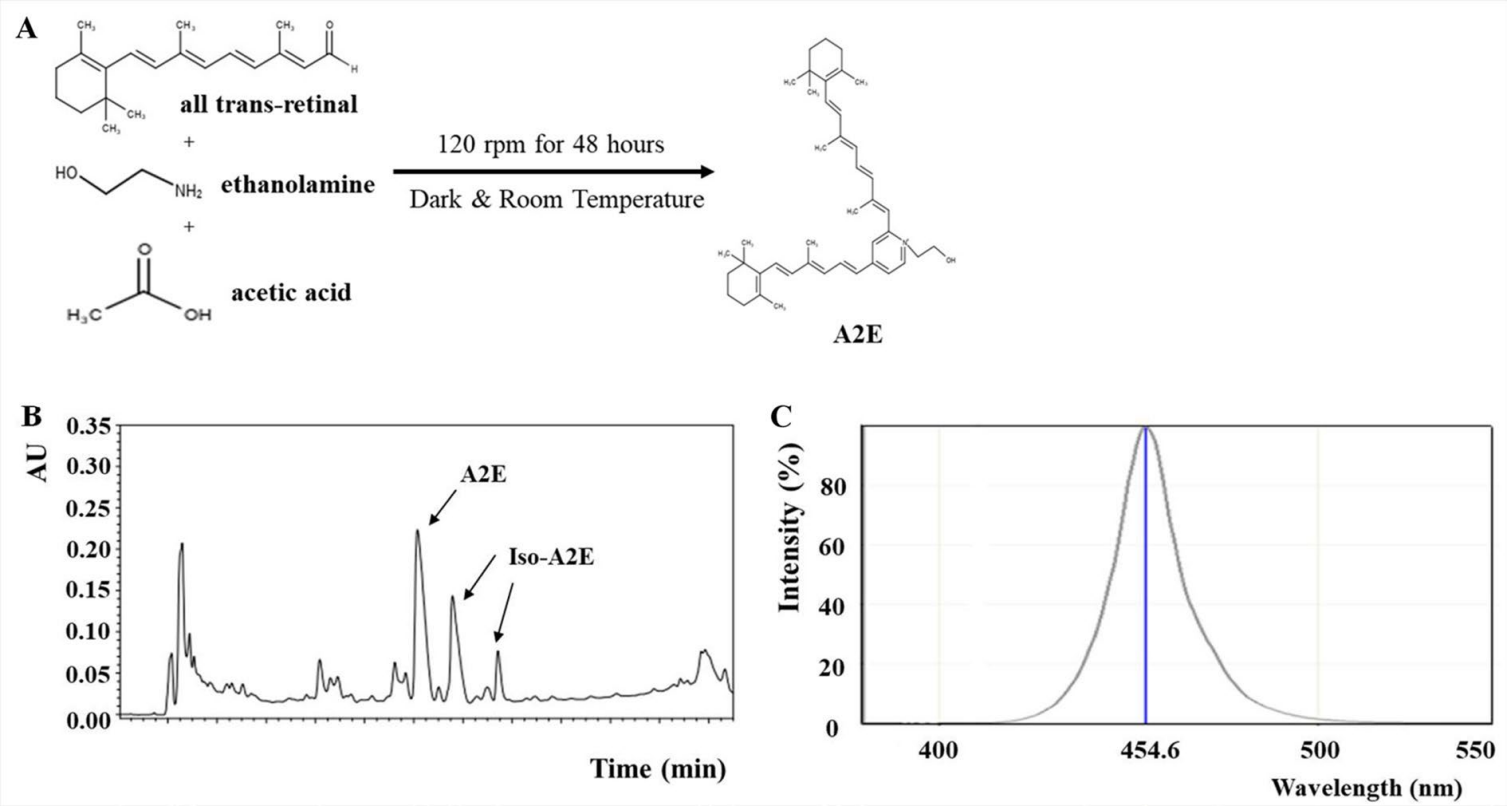
N-retinylidene-N-retinyl-ethanolamine (A2E) synthesis and the light intensity of the manufactured blue light transilluminator. (**A**) The scheme of A2E synthesis. (**B**) The result of A2E synthesis. Synthetic A2Es consisted of one A2E and two A2E isotypes. (**C**) The intensity of the blue light transilluminator. The peak wavelength of the irradiated light was 454.6 nm, with the majority of light falling within the range of blue light.

A2E was synthesized according to Parish’s method^27^. Briefly, all-trans-retinal (100 mg, 352 mM) and 9.5 mg of ethanolamine (155 mM) were dissolved in 3.0 mL of ethanol (EtOH). Then, 9.3 µL of acetic acid (155 mM) was added, and the mixture was reacted at 120 rpm at room temperature in the dark for 48 h (Fig. 1A). To evaluate the purity of the synthetic A2E, liquid chromatography was conducted using a Waters Alliance 2695 HPLC system with a photodiode array detector. The analytic conditions are described in Table 1.

**Table 1:** Analysis condition for synthetic N-retinylidene-N-retinylethanolamine (A2E).

	Parameters	Conditions	
	Column	Zorbax extended—C18 (C18, 4.6 mm × 150 mm, 5 µm)	
	Flow rate	1 mL/min	
	Injection volume	10 µL	
	UV detection, Run time	380 nm, 25 min	
	Run time	25 min	

	Time (min)	% Acetonitrile	% 0.1%trifluoroacetic acid
Gradient	0	85	15
	3	85	15
	22	96	4
	23	100	0
	24	85	15

### Manufacturing and intensity measurement of blue light transilluminator

The blue light emitting diodes (LEDs, Sumbulbs, 12 V Blue Light, Shenzhen, China) were used for the blue light transilluminator. The light intensity and wavelength were tested using the NeoLight PL5000 (PIMACS, Kyunggi, Korea), the Programmable AC Power Source 61,052 (Chroma ATE Inc., Taoyuan, Taiwan), and the Voltech PM3000A Universal Power Analyzer (Advanced Test Equipment Corp. CA, USA) by Korea Photonic Technology Institute (KOPTI, Gwangju, Korea). The analytical condition was maintained at 25.1 ± 0.6 °C and 51.8 ± 1.5% relative humidity.

### In vitro study schedule including sample collecting

**Figure 2:**
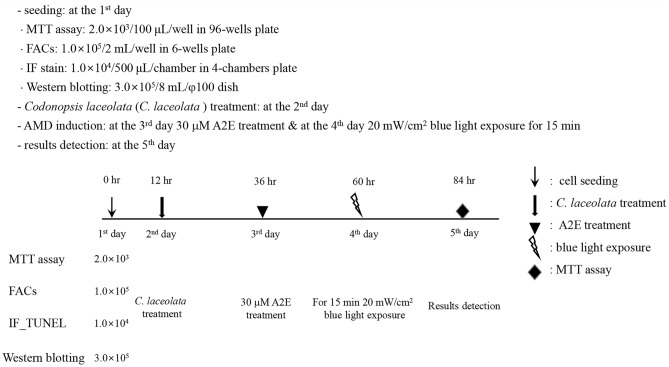
Cell culture protocols depending on the analysis and sample harvest time.

In this study, several cell culture protocols were utilized (Fig. 2). The 3-[4,5-dimethylthiazole-2-yl]-2,5-diphenyltetrazolium bromide (MTT) assay (VWR Life Science, OH, USA, 76,022–152) was performed to assess cell viability. Fluorescence activated cell sorting (FACS) analysis was conducted to determine the population of apoptotic cells. The immunofluorescence (IF) assay was used to analyze terminal deoxynucleotidyl transferase dUTP nick end labeling (TUNEL) staining for apoptotic cell status, as well as A2E auto-fluorescence and activation of the anti-oxidative system, Kelch-like ECH-associated protein 1 (Keap1)/nuclear factor erythroid-2-related factor 2 (Nrf2)/heme oxygenase-1 (HO-1) pathway. Western blotting was performed to examine the apoptotic pathway (B cell lymphoma 2 (Bcl-2) family), mitogen-activated protein kinase (MAPK), and indicators of oxidative stress (Keap1/Nrf2/HO-1) and carbonyl stress (4-hydroxynonenal (4-HNE)). Figure 6 provides a detailed description of the specific parameters for each assay.

### Analysis of the accumulation and cytotoxicity of A2E in ARPE-19 cells

The adult retinal pigment epithelium (ARPE-19) cell line was purchased from the American Type Culture Collection (ATCC, Manassas, VA, USA, CRL-2302™) and maintained in Dulbecco’s modified eagle medium (DMEM, Gibco, 12,100,046) containing 10% fetal bovine serum (FBS, Gibco, Waltham, MA, USA, 30,067,334) and 1% penicillin. To evaluate the level of A2E accumulation in ARPE-19 cells, a confocal laser scanning microscope (Nanoscope systems, K1-Fluo, Daejeon, Korea) was used. The nucleus was counter-stained with 4',6-diamidino-2-phenylindole (DAPI, Thermo Fisher, Waltham, MA, USA, 62,247). A range of A2E concentrations from 0 to 40 μM was used and confirmed at 488 nm.

To measure the cytotoxicity of synthetic A2E on ARPE-19 cells, the cells were seeded at a density of 2 × 10^3^ cells per well. After 36 h, various doses of A2E (0, 10, 20, 30, and 40 μM) were administered. Then, 24 h after A2E treatment, three levels of blue light intensity (0, 20, and 40 mW/cm^2^) were applied for 15 min. After 24 h, an MTT assay was conducted to evaluate the cytotoxicity of A2E and/or blue light in ARPE-19 cells, and the results were obtained using a Multiskan SkyHigh Microplate Spectrophotometer (Thermo Fisher Scientific, Waltham, MA, USA).

### The analysis of apoptosis pathway of ARPE-19 cells by A2E and/or blue light

The abundance of apoptotic ARPE-19 cells depending on the dose of synthetic A2E and/or the intensity of blue light was measured using IF, TUNEL staining, and FACS analysis. Based on the MTT assay and A2E accumulation, the doses of A2E and the intensity of blue light were confirmed as 0, 30, or 40 μM A2E and 0, 20, or 40 mW/cm^2^ blue light.

For the FACS analysis, Invitrogen Alex Fluor 488 apoptosis detection kit (Invitrogen, Waltham, MA, USA, R37174) was used. The cells were harvested using trypsin (Gibco, 25,200,056). Single cells were washed, stained using Alex Fluor 488 and propidium iodide from the kit, and the results were acquired using Guava Easycyte (Millipore, Burlington, MA, USA).

For the TUNEL assay, the Click-iT™ Plus TUNEL assay kit (Invitrogen, C10618) was used. A2E cells were treated under the same conditions regarding A2E dose and/or blue light intensity. The media was discarded, and the attached cells were washed with phosphate buffered saline (PBS, Lonza, Walkersville, MD, USA, BE17-517Q). The cells were then fixed with 4% formaldehyde (Daejung, Siheung-si, Gyeonggi-do, Korea, P2031) in PBS. After fixation, 0.25% Triton X-100 (Sigma-Aldrich, Saint Louis, MO, USA, 9036–19-5) in PBS was added, and the cells were stained following the manufacturer’s guidelines. Finally, the images were obtained using a K1-Fluo confocal microscope (Nanoscope systems).

### The analysis of carbonyl stress and oxidative stress on ARPE-19 cells by A2E and/or blue light

Carbonyl stress and oxidative stress were assessed using western blotting. To evaluate the carbonyl stress induced by A2E and/or blue light, the representative carbonyl stress-induced metabolite, 4-HNE (abcam, Ab46545), was analyzed. Oxidative stress analysis was conducted using primary antibodies for Nrf2 (Invitrogen, PA5-27,882) and HO-1 (Invitrogen, PA5-77,833).

### Preparation and analysis of C. *lanceolata* extract and active compounds

The root of *C. lanceolata* was obtained from Wellphyto Co. (Gwangju, Korea) and deposited at Mokpo National University (MNUCSS-CL-01). To extract the active compounds, 20 g of dried *C. lanceolata* root was subjected to hot water extraction at 100 ℃ for 4 h. The resulting extract was freeze-dried. In our previous study^28^, lobetylin was confirmed as an active compound in *C. lanceolata*. This study was conducted to comply with the IUCN Policy Statement on Research involving Species at Risk of Extinction and the Convention on the Trade in Endangered Species of Wild Fauna and Flora.

### Analysis of the anti-cytotoxic and anti-apoptotic effects of C. *lanceolata* in an in vitro model of AMD

All in vitro experiments were performed as shown in Fig. 2. To determine the proper concentration of *C. lanceolata* for this study, five doses of *C. lanceolata* (5, 10, 25, 50, and 100 μM) were tested, along with a normal control group (CON; 0 μM A2E and 20 mW/cm^2^ blue light), an induction control group (A2E; 30 μM A2E and 20 mW/cm^2^ blue light), and a positive control group (Lutein; 30 μM A2E, 20 mW/cm^2^ blue light, and 25 μM lutein). At 12 h after seeding ARPE-19 cells in a 96-well plate (2 × 10^3^ cell/well), the reagents were administered. At 24 h after *C. lanceolata* treatment, A2E was added to each well, excluding the normal control group. At 24 h after A2E addition, each well was irradiated with 20 mW/cm^2^ blue light for 15 min. Finally, 24 h after blue light irradiation, the MTT assay was conducted to evaluate the anti-cytotoxic effect of *C. lanceolata*.

The anti-apoptotic effect of *C. lanceolata* was measured using IF for TUNEL staining and FACS analysis. The experimental groups included the CON, A2E, Lutein, and three doses of *C. lanceolata* (10, 25, 50 μg/mL) groups. A2E was applied 24 h after seeding the ARPE-19 cells, and blue light was irradiated after 48 h and after 72 h. The cells were harvested using trypsin to evaluate apoptosis. The subsequent steps were the same for IF, TUNEL, and FACS analyses.

### Analysis of suppression effect of C. *lanceolata* on three stresses

The anti-apoptotic mechanism of the Bcl-2 family pathway was evaluated using primary antibodies against Bcl-2 (Santa Cruz Biotechnology, sc-7382), B-cell lymphoma-extra large (Bcl-XL, Invitrogen, PA5-17805), and Bcl-2 interacting mediator of cell death (Bim, Santa Cruz Biotechnology, sc-374358). The results of were obtained using Davinch-Western^TM^ (Davinch-K, Seoul, Korea).

To evaluate the anti-apoptotic effect of *C. lanceolata*, the changes in the Keap1/Nrf2/HO-1 pathway and 4-HNE levels were assessed using western blotting and IF. Primary antibodies for Keap1 (Invitrogen, PA5-99,434), Nrf2 (Invitrogen, PA5-27,882), HO-1 (Invitrogen, PA5-77,833), and 4-HNE (abcam, Ab46545) were used.

### Statistics

The results are expressed as the mean ± standard deviation (SD). Following one-way analysis of variance (ANOVA) analysis, Dunnett’s multiple comparison was conducted. Differences with *p* values below 0.05 were considered significant.

## Results

### A2E synthesis and the light wavelength of a self-manufactured transilluminator

As shown in Fig. 1A, 352 mM all-trans-retinal and 155 mM ethanolamine were dissolved in 3.0 mL of EtOH. Then, 155 mM acetic acid was added to the mixture, and they were reacted through centrifugation at 120 rpm at room temperature in the dark for 48 h (Fig. 1A). After the reaction, three types of A2E were synthesized and verified using liquid chromatographic analysis. One A2E was found approximately at 12 min (retention time), while two A2E isotypes (Iso-A2Es) were found around 14 min and 16 min (Fig. 1B). The analytical conditions are presented in Table 1. The most effective light for AMD induction is blue light, which has a wavelength range of 400–500 nm^29^. The self-manufactured blue light transilluminator used in this study mainly emitted blue light within this range, with a peak of the wavelength of 454.6 nm (Fig. 1C).

### A2E permeated into ARPE-19 cells and the population of dead cells increased depending on both A2E and blue light

**Figure 3:**
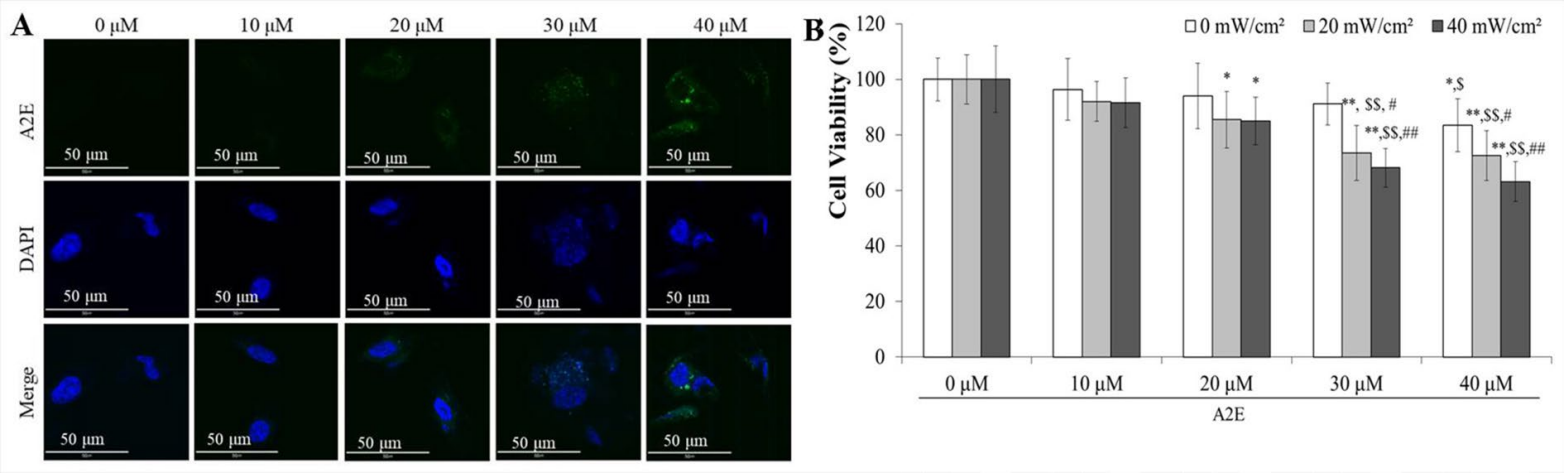
Cytotoxicity and A2E accumulation in adult retinal pigment epithelial (ARPE)-19 cells, depending on the dose of A2E and the intensity of blue light. (**A**) A2E accumulation in the cytoplasm of ARPE-19 cells depending on the dose of A2E. A clear presence of A2E in the cytoplasm was observed, particularly at a dose of 20 μM A2E. Scale bar, 50 μm. Magnification, 1000 × . (**B**) Cell viability of ARPE-19 cells according to the dose of A2E and/or blue light intensity. *N* = 6. The results are presented as the mean ± standard deviation (SD). ^*^, *p* < 0.05; ^**^, *p* < 0.001 compared to 0 μM A2E. ^$^, *p* < 0.05; ^$$^ *p* < 0.01 compared to 10 μM A2E. ^#^, *p* < 0.05; ^##^, *p* < 0.001 compared to 20 μM A2E.

A2E, when reacted with blue light, induces AMD and exhibits autofluorescence^30^. To evaluate the level of A2E permeation in the cytoplasm, an IF assay was performed after treatment with 0, 10, 20, 30, or 40 μM A2E. In treatments with 10 μM A2E or less, the green color was unclear. However, in treatments with 20 μM A2E or more, green spots could be clearly detected in the cytoplasm (Fig. 3A). A2E decreased cell viability from 100 ± 7.75% in the 0 μM treatment group to 83.4 ± 9.50% in the 40 μM treatment group (Fig. 3B and Table 2) without blue light treatment. Co-treatment with A2E and blue light dose-dependently increased cytotoxicity. Under 20 mW/cm^2^ blue light illumination, the viabilities in the 30 μM and 40 μM A2E treatment groups were 73.5 ± 9.91% and 72.5 ± 8.89%, respectively. Under 40 mW/cm^2^ blue light exposure, the viabilities in the 30 μM and 40 μM A2E treatment groups were only 68.1 ± 6.96% and 63.2 ± 7.17%, respectively. Based on these results, the optimal condition was determined to be 30 μM A2E treatment with 20 mW/cm^2^ blue light illumination.

**Table 2:** The results of cytotoxicity in adult retinal pigment epithelial (ARPE)-19 cells, depending on the dose of A2E and the intensity of blue light (*N* = 6).

		Blue light		
		0 mW/cm^2^	20 mW/cm^2^	40 mW/cm^2^
A2E	0 μM	100.0 ± 7.75	100.0 ± 8.86	100.0 ± 11.93
	10 μM	96.4 ± 11.14	92.0 ± 7.17	91.6 ± 8.93
	20 μM	94.1 ± 11.78	85.5 ± 10.19*	85.0 ± 8.57*
	30 μM	91.1 ± 7.58	73.5 ± 9.91**^,$$,##^	68.1 ± 6.96**^,$$,#^
	40 μM	83.4 ± 9.50*^,$^	72.5 ± 8.89**^,$$,##^	63.2 ± 7.17**^,$$,##^

The results are presented as the mean ± standard deviation (SD). *, *p* < 0.05; **, *p* < 0.001 compared to 0 μM A2E. ^$^, *p* < 0.05; ^$$^ *p* < 0.01 compared to 10 μM A2E. ^#^, *p* < 0.05; ^##^, *p* < 0.001 compared to 20 μM A2E.

### A2E and blue light induces apoptosis in ARPE-19 cells via Nrf2/HO-1 pathway and carbonyl stress

**Figure 4:**
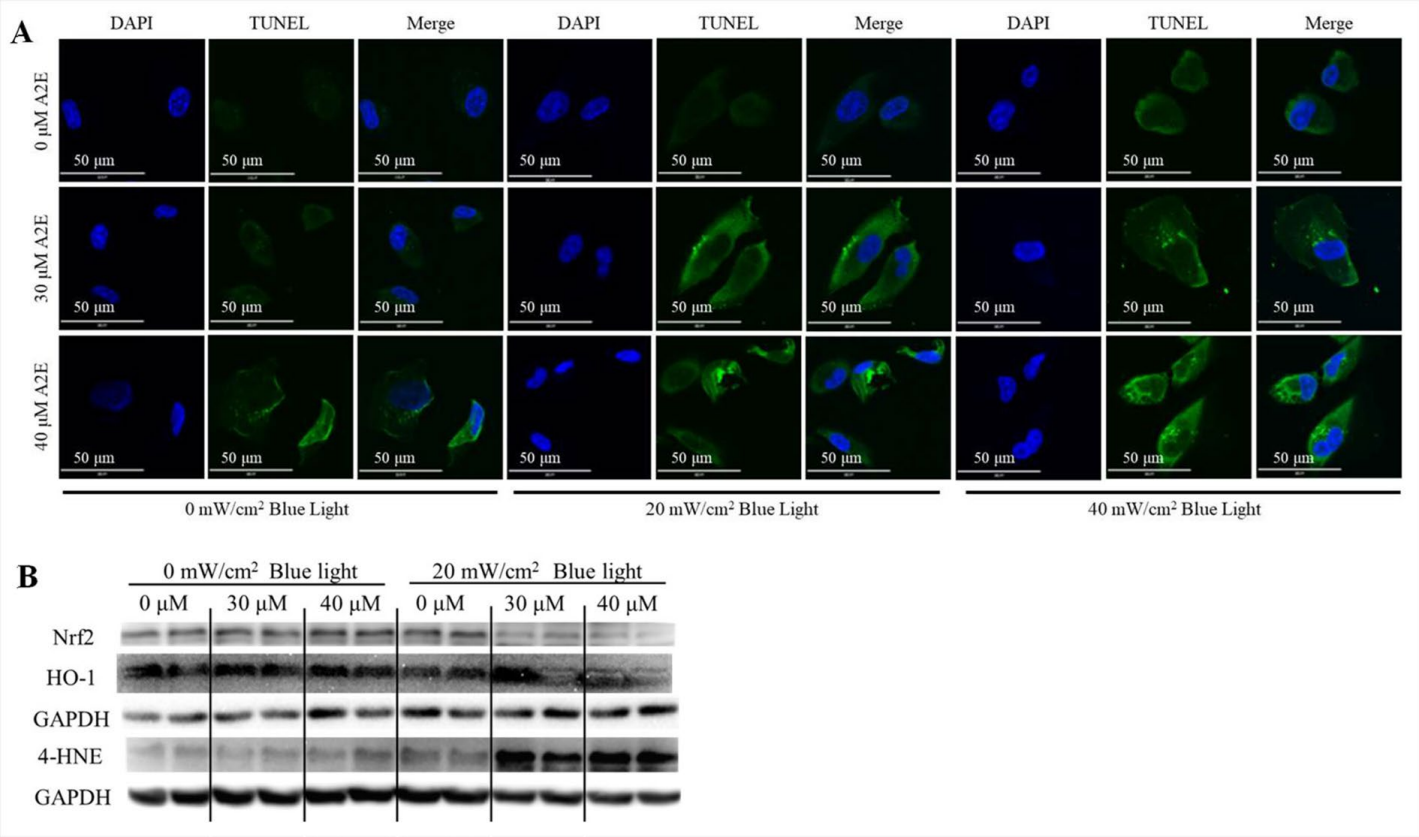
Apoptosis, oxidative stress, and carbonyl stress in ARPE-19 cells depending on the level of A2E and the intensity of blue light. (**A**) The results of the TUNEL assay for ARPE-19 cell death at various doses of A2E and/or blue light intensities. Scale bar, 50 μm. Magnification, 1000 × . (**B**) The results of apoptosis-related pathway analysis. *N* = 3.

To analyze the apoptotic effect of A2E and/or blue light treatment, TUNEL assay was performed followed by IF analysis (Fig. 4A). The results were similar to those observed in cell viability tests (Fig. 3B). A2E treatment dose-dependently increased the number of apoptotic cells. Under 20 mW/cm^2^ blue light illumination, 30 μM or 40 μM A2E treatment induced cell apoptosis, particularly resulting in irregular cell morphology. Similar results were obtained with 40 mW/cm^2^ blue light treatment. To confirm cell death caused by A2E and blue light, FACS analysis was conducted (Table 3). A2E treatment alone dose-dependently increased the population of dead cells, including necrotic cells, from about 5.1 ± 0.68% in the 0 μM A2E treatment group to 23.4 ± 3.50% in the 40 μM A2E treatment group. Co-treatment with A2E and blue light also dose-dependently increased the number of dead cells. Specifically, after 30 μM A2E treatment with 20 mW/cm^2^ blue light exposure, the population of apoptotic cells was 21.6 ± 0.072%. Accordingly, this condition was selected as the AMD cell model. Although there are many factors that induce cell death, oxidative and carbonyl stress are among the important ones^31^. To examine the effect of treatment with A2E, blue light, or their combination on oxidative and carbonyl stress, the levels of oxidative stress markers, such as the Nrf2/HO-1 pathway and the carbonyl stress marker 4-HNE were measured (Fig. 4B). Without blue light treatment, an upregulation of 4-HNE due to A2E treatment was observed. However, under 20 mW/cm^2^ blue light treatment, A2E treatment suppressed the Nrf2/HO-1 pathway but increased the level of 4-HNE in a dose-dependent manner.

**Table 3:** The results of FACs analysis for ARPE-19 cells’ apoptosis according to A2E dose and/or blue light intensity.

	0 mW/cm^2^ Blue Light			20 mW/cm^2^ Blue Light			40 mW/cm^2^ Blue Light		
A2E	0	30	40	0	30	40	0	30	40
Normal (%)	94.9 ± 0.62	77.9 ± 1.08**	76.5 ± 2.95**	93.1 ± 0.23	77.5 ± 0.67^#^	72.8 ± 6.46^@^	80.1 ± 8.06	72.8 ± 6.46	70.1 ± 4.82
Necrosis (%)	3.6 ± 0.43	21.3 ± 1.30**	23.0 ± 3.21**	0.9 ± 0.31	0.8 ± 0.05	1.9 ± 1.11	7.8 ± 6.84	1.9 ± 1.11	12.0 ± 5.70
Early apoptosis (%)	0.9 ± 0.23	0.6 ± 0.17	0.5 ± 0.25	1.6 ± 0.78	4.5 ± 0.31^#^	7.3 ± 1.92^@^	2.6 ± 0.72	7.3 ± 1.92^&^	7.8 ± 5.53
Late apoptosis (%)	0.6 ± 0.22	0.1 ± 0.04	0.1 ± 0.04^$^	4.4 ± 0.71	17.1 ± 0.41^#^	18.0 ± 5.84	9.4 ± 1.85	18.0 ± 5.84	10.1 ± 4.81
Total apoptosis (%)	1.5 ± 0.25	0.8 ± 0.21*	0.5 ± 0.28^$^	5.9 ± 0.08	21.6 ± 0.72^#^	25.3 ± 5.35^@^	12.0 ± 2.28	25.2 ± 5.35^&^	17.9 ± 0.95^%^
Necrosis + apoptosis (%)	5.1 ± 0.62	22.1 ± 1.08**	23.5 ± 2.95^$$^	6.9 ± 0.23	22.5 ± 0.67^#^	27.2 ± 6.46^@^	19.9 ± 8.06	27.2 ± 6.46	29.9 ± 4.82

In 20 mW/cm^2^ blue light irradiated groups A2E dose-dependently increased the apoptotic cells (*N* = 3). The results were described the mean ± standard deviation (SD). * p < 0.05 vs. 0 mW/cm^2^ blue light and 0 μM A2E; ** *p* < 0.001 vs. 0 mW/cm^2^ blue light and 0 μM A2E; $ *p* < 0.05 vs. 0 mW/cm^2^ blue light and 30 μM A2E; # *p* < 0.05 vs. 20 mW/cm^2^ blue light and 0 μM A2E; @ *p* < 0.05 vs. 20 mW/cm^2^ blue light and 30 μM A2E; & *p* < 0.05 vs. 40 mW/cm^2^ blue light and 0 μM A2E; % *p* < 0.001 vs. 40 mW/cm^2^ blue light and 30 μM A2E.

### C. *lanceolata* prevented cell death induced by A2E and blue light in a dose-dependent manner

**Figure 5:**
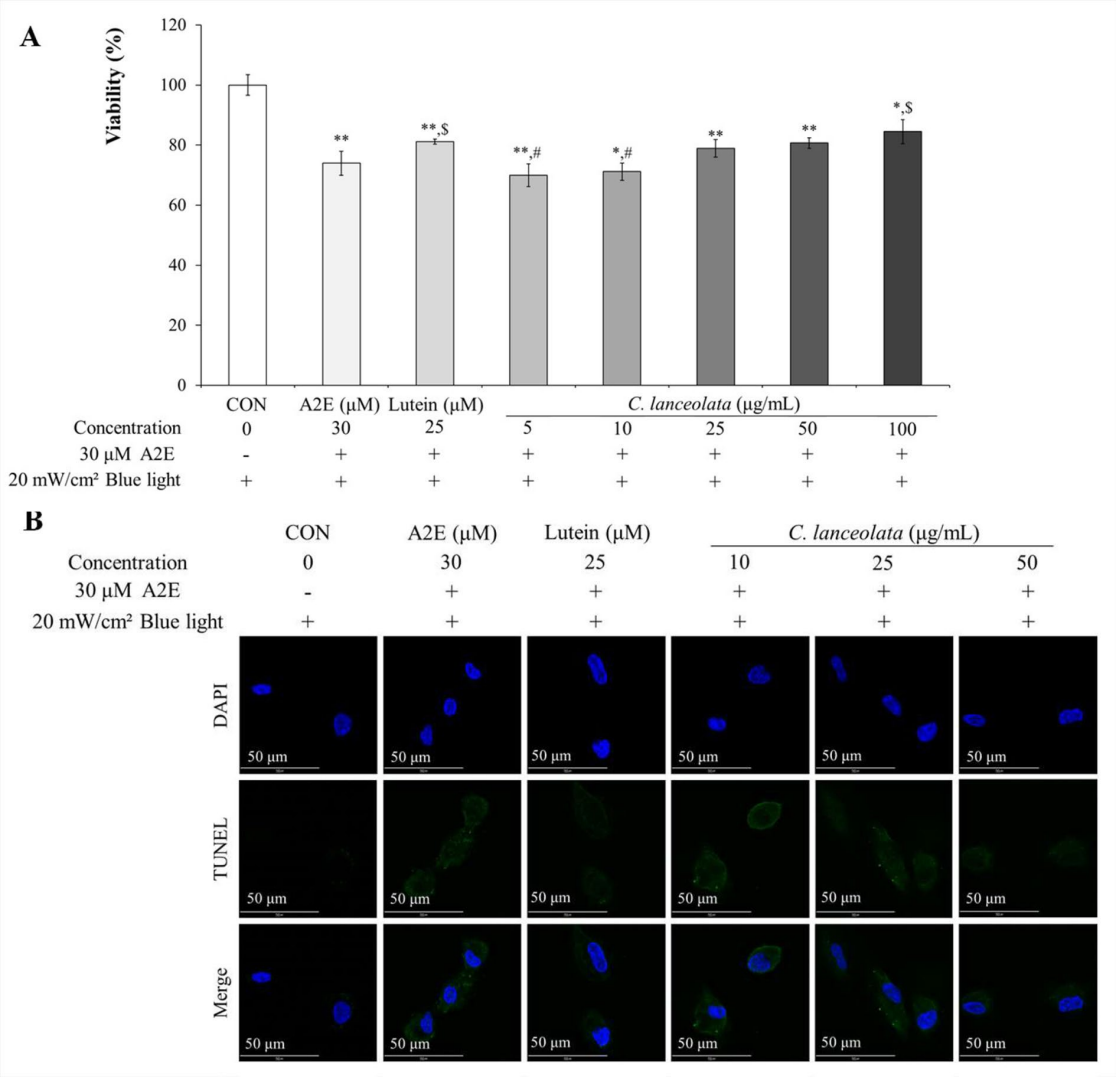
*Codonopsis lanceolata* prevented cell death induced by A2E and blue light. (**A**) *C. lanceolata* dose-dependently ameliorated cell death induced by A2E and blue light (*N* = 3). ^*^, *p* < 0.05; ^**^, *p* < 0.001 compared to CON; ^$^, *p* < 0.05 compared to the A2E and blue light treatment group; ^#^, *p* < 0.05 compared to the lutein treatment group. (**B**) *C. lanceolata* dose-dependently suppressed RPE-19 cell apoptosis induced by A2E and blue light (green spot). Scale bar, 50 μm. Magnification, 1000 × .

To measure the preventive effect of *C. lanceolata* against cytotoxicity induced by A2E and blue light, an MTT assay was performed (Fig. 5A). Co-treatment with 30 μM A2E and 20 mW/cm^2^ blue light increased the population of dead ARPE-19 cells, but 25 μM lutein treatment prevented their cytotoxicity. Although only in the 100 μg/mL *C. lanceolata* treatment group, the live cell percentage was similar to that in the 25 μM lutein treatment group, *C. lanceolata* treatment resulted an increasing trend of live cell population depending on the dose. A2E and blue light co-treatment significantly induced cell death compared to the control, but *C. lanceolata* treatment dose-dependently decreased the level of apoptosis (green) caused by A2E and blue light co-treatment (Fig. 4B). To confirm the result of the IF assay (Fig. 5B), the anti-apoptotic effect of *C. lanceolata* was analyzed using FACS (Table 4). Compared to the result in the control group (the population of total apoptotic cells, 5.8 ± 2.37%), A2E and blue light co-treatment induced a higher amount of cell death (10.4 ± 2.90%), but *C. lanceolata* treatment dose-dependently prevented A2E and blue light-induced cell death. In particular, the population of dead cells in the 50 μg/mL *C. lanceolata* treatment group (5.0 ± 1.83%) was similar to that in the control group. Compared to the population of normal cells in the 25 μM lutein treatment group (82.2 ± 0.95%), the population in the 25 μg/mL *C. lanceolata* treatment group was 83.5 ± 2.03%, indicating that the normal cell percentage in the lutein treatment group was similar to that in the 25 μg/mL *C. lanceolata* treatment group.

**Table 4:** The population percentage of the apoptotic cells.

	CON	A2E (μM)	Lutein (μM)	*C. lanceolata* (μg/mL)		
Concentration	0	30	25	10	25	50
30 μM A2E	−	+	+	+	+	+
20 mW/cm^2^ Blue light	+	+	+	+	+	+
Normal (%)	91.3 ± 1.43	79.1 ± 2.88*	82.2 ± 0.95**	80.8 ± 1.01**	83.5 ± 2.03*	84.7 ± 2.24*^,@^
Necrosis (%)	2.9 ± 0.99	10.5 ± 0.52**	13.3 ± 1.34**^,$^	10.4 ± 1.47*	10.0 ± 0.56**^,#^	10.3 ± 0.55**^,#^
Early apoptosis (%)	3.8 ± 1.97	7.0 ± 2.08	2.5 ± 0.39^$^	5.0 ± 0.70^#^	4.0 ± 1.10	3.1 ± 1.37
Late apoptosis (%)	2.0 ± 0.40	3.4 ± 0.82	2.0 ± 0.14^$^	3.8 ± 0.35*^,#^	2.2 ± 0.73^@,&^	1.9 ± 0.58^@,&^
Total apoptosis (%)	5.8 ± 2.37	10.4 ± 2.90**	4.5 ± 0.39^$^	8.8 ± 0.54^##^	6.2 ± 1.66	5.0 ± 1.83^@,&^

*C. lanceolata* dose-dependently prevented A2E and blue light-induced ARPE-19 cells’ death (*N* = 3). *p < 0.05 vs. CON; **p < 0.001 vs. CON; ^$^*p* < 0.05 vs. A2E and blue light treatment group; ^#^*p* < 0.05 vs. lutein treatment group; ^##^*p* < 0.001 vs. lutein treatment group; ^@^*p* < 0.05 vs. 10 μg/mL *C. lanceolata* treatment group; ^&^*p* < 0.05 vs. 25 μg/mL *C. lanceolata* treatment group.

### C. *lanceolata* prevented apoptosis in ARPE-19 cells by increasing Bcl-2 and Bcl-XL and decreasing Bim, stimulating the activation of the Keap1/Nrf2/HO-1, and downregulating 4-HNE

**Figure 6:**
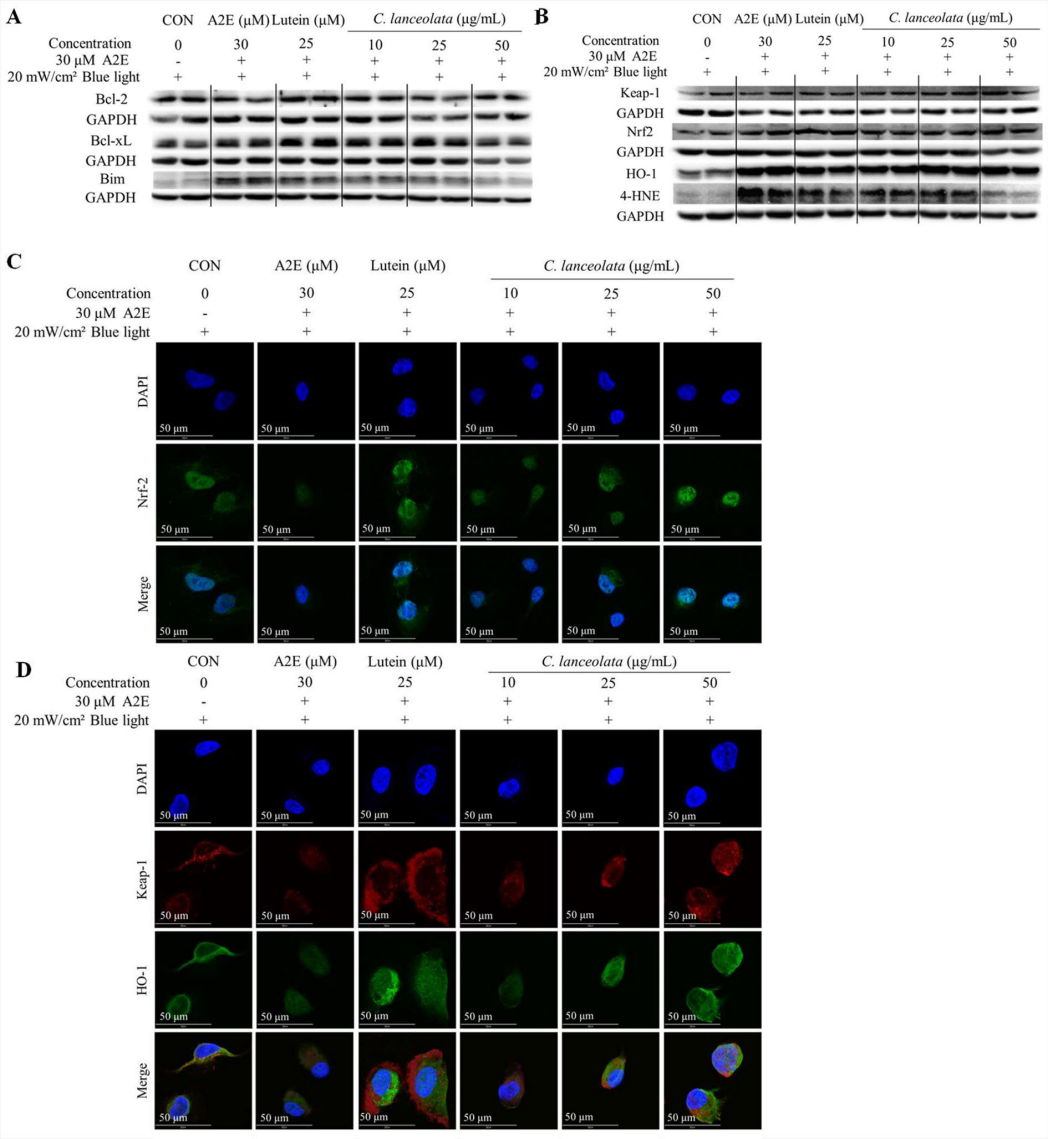
*Codonopsis lanceolata* regulated the apoptosis of ARPE-19 cells through the activation of the Keap1/Nrf2/HO-1 pathway and the suppression of 4-HNE. (**A**) *C. lanceolata* modulated the Bcl-2 family, increasing the levels of the antiapoptotic proteins Bcl-2 and Bcl-XL, while decreasing the level of the proapoptotic protein Bim (*N* = 3). (**B**) *C. lanceolata* treatment activated the anti-oxidative Keap1/Nrf2/HO-1 pathway but decreased the expression of 4-HNE in a dose-dependent manner (*N* = 3). (**C**) *C. lanceolata* treatment stimulated the expression of nuclear Nrf2, which functions as a transcription factor for HO-1 (green spots). (D) *C. lanceolata* treatment dose-dependently induced the expressions of Keap1 and HO-1. Scale bar, 50 μm. Magnification, 1000 × .

The results shown in Fig. 5 suggested that *C. lanceolata* prevented apoptosis in ARPE-19 cells, which was caused by co-treatment with A2E and blue light. The Bcl-2 family includes anti-apoptotic proteins, such as Bcl-2 and Bcl-xL, as well as the pro-apoptotic protein Bim^32^. We analyzed the Bcl-2 family proteins to investigate the anti-apoptotic mechanism of *C. lanceolata* (Fig. 6A). Co-treatment with A2E and blue light regulated the levels of Bcl-2 family proteins by downregulating Bcl-2 and Bcl-XL and upregulating Bim. However, *C. lanceolata* suppressed this effect in a dose-dependent manner, increasing Bcl-2 and Bcl-XL levels while decreasing Bim expression (Fig. 6A). Keap1/ Nrf2/ HO-1 pathway plays an important role in the anti-oxidative system. Under normal conditions, Keap1 and Nrf2 are bound together in the cytoplasm. However, when cells are stressed, Nrf2 separates from Keap1 and translocates to the nucleus, acting as a transcription factor for HO-1^33^. *C. lanceolata* increased the expression levels of Keap1, Nrf2, and HO-1 proteins in a dose-dependent manner and effectively reduced the level of 4-HNE (Fig. 6B). To confirm the activation of the Keap1/Nrf2/HO-1 pathway, an IF assay was performed (Fig. 6C and D). In the A2E group, the expression level of Nrf2 in the nucleus was lower compared to the control group. However, similar to lutein treatment, *C. lanceolata* treatment dose-dependently increased the expression of Nrf2 in the nucleus (Fig. 6C). Similarly, the expression of Keap1 and HO-1 were dose-dependently increased in the cytoplasm (Fig. 6D), consistent with the results of western blotting in the *C. lanceolata* treatment group. Overall, these results indicate that *C. lanceolata* effectively activated the Keap1/Nrf2/HO-1 pathway.

## Discussion

AMD is one of the leading causes of blindness worldwide^1^, and it is closely associated with aging. The global population of aging individuals is rapidly increasing^4^. According to the stage of AMD, medications can be divided into two types: treatments for atrophic and neovascular AMD. The therapeutic method for neovascular AMD includes laser or anti-VEGF treatment, which aims to inhibit angiogenesis in the choroidal area^10^. The purpose of atrophic AMD treatment is to prevent the death of RPE cells and is associated with eliminating various factors-related to apoptosis, such as oxidative stress, inflammation, and carbonyl stress. Current medications have various adverse effects such as macular edema, retinovascular disease, hypertension, insulin resistance, insomnia, skin thinning, gastric ulceration, eye bleeding, and increased risk of infection^11–14^. Due to these adverse effects, there is a need for new AMD drugs based on the type of AMD such as atrophic or neovascular AMD that are both more effective and safer. For successful drug development, it is crucial to establish an appropriate AMD model system.

Depending on the time of occurrence and angiogenesis in the choroidal area, AMD can be classified into two types: atrophic and neovascular AMD. The main difference between them is that atrophic AMD is caused by the death of RPE cells in the early stage. While neovascular AMD occurs due to angiogenesis in the choroidal area after RPE cell death^9^. The initiation of AMD is believed to involve the death of RPE cells, which can be caused by oxidative stress, inflammation, and carbonyl stress^31^. There are various reactive oxygen species (ROS), including hydroxyl radical (OH^·^), hydroxide ion (HO^-^), triplet oxygen (^3^O_2_), superoxide anion (O_2_^·−^), peroxide ion (O_2_^2−^), hydrogen peroxide (H_2_O_2_), and nitric oxide (NO^·^)^34^. The ROS related to AMD occurrence have been identified as OH^·^^35^, O_2_^·−^^36^, and H_2_O_2_^37^. However, the relationship between NO^·^ levels and AMD occurrence is unclear, as controversial results exist, such as upregulation^38^ and down-regulation^39^ in AMD patients. Oxidative stress and inflammation are deeply related to the development of several pathologies^34^, as inflammatory cells stimulate oxidative stress^40^ and ROS induce an increase in proinflammatory gene expression^41^. The Keap1/Nrf2 ARE is one of the representative anti-oxidative systems. It can control not only oxidative stress but also inflammation through the NF-κB pathway. This pathway is suggested as a good therapeutic target against many diseases, such as cancer, diabetes, inflammatory disease, and respiratory disease^42,43^. Carbonyl stress refers to the negative process in several parts of the bio-organism, such as protein, plasma membrane, and DNA, that is caused by carbonyl compounds generated through non-oxidative mechanisms^44^. Some carbonylation-related metabolites, such as 4-HNE^45^, *trans*-4-hydroxy-2-hexenal (4-HHE)^46^, and malondialdehyde (MDA)^47^, stimulate the occurrence of AMD. Apoptosis can be classified into extrinsic and intrinsic pathways^32^. The extrinsic pathway is initiated by the interaction of transmembrane receptors and ligands, such as tumor necrosis factor receptor 1 (TNFR1)/TNF-α, FasR/FasL, and death receptors (DRs)/tumor necrosis-related apoptosis-inducing ligand (TRAIL)^48–50^. The intrinsic pathway is deeply connected to mitochondrial changes, which can be positively or negatively controlled by Bcl-2 family proteins but is not related to death receptors^51^. Bcl-2 family proteins include anti-apoptotic proteins, such as Bcl-2, Bcl-extra (Bcl-x), and Bcl-xL, and pro-apoptotic proteins, such as Bcl-2 associated X (Bax), Bcl-2 antagonist/killer 1 (Bak), and Bim.

The atrophic AMD is initiated by the death of RPE cells, which is caused by oxidative stress, inflammation, and carbonyl stress. Although there are many risk factors, such as aging, tobacco smoking, cataract surgery, family history, and overweight, the proximate and major factor is believed to be the epoxide type of A2E, which is produced by A2E and blue light^6^. However, it is unclear what level of A2E and intensity of blue light are required to induce an in vitro AMD model. From various studies, the level of A2E has been reported to range from 20 to 100 μM, and the intensity of blue light has been tested from 0.04 to 35 mW/cm^2^^15–17^. To investigate similar conditions in bio-organisms with atrophic AMD, we evaluated the permeability of A2E to the cytoplasm and the relationship between RPE cell death and A2E level/blue light intensity (Figs. 3 and 4). A2E was found to pass through the cell membrane in a dose-dependent manner (Fig. 3A), and its cytotoxic effect on ARPE-19 cells increased with the level of A2E in the cytoplasm and the intensity of blue light (Fig. 3B). The population of apoptotic cells was increased in a dose-dependent manner with A2E and blue light treatment, but the number of necrotic cells also increased (Fig. 4A and Table 3). Taking into account both the number of apoptotic and necrotic cells, the combination of 30 μM A2E treatment and 20 mW/cm^2^ blue light transillumination was selected as the best in vitro AMD model condition. To confirm the apoptosis-related oxidative stress and carbonyl stress pathway, several factors were analyzed. Under 30 μM A2E treatment and 20 mW/cm^2^ blue light transillumination, the Nrf2/HO-1 anti-oxidative system was suppressed and the level of 4-HNE was increased in ARPE-19 cells (Fig. 4B). These results indicate that ARPE-19 cell death is induced by Nrf2/HO-1 anti-oxidative system-related oxidative and carbonyl stress (Figs. S1, S3, S3) .

While developing potential synthetic chemicals against atrophic AMD is an option, natural products that have been used as culinary and/or medicinal materials for a long time can be another alternative, as they may be relatively safe if they have been consumed as edible substances for a prolonged period. However, before considering them as candidates, their therapeutic mechanisms, such as anti-oxidation, anti-inflammation, and/or anti-carbonylation for preventing RPE cell death, should be confirmed. *C. lanceolata* is a culinary/medicinal material that has been used for a long time in Asia and has been reported to have biological effects, such as anti-hepatitis^24^, anti-inflammatory^25^, and anti-asthmatic effects^26^. These observations suggest that *C. lanceolata* has anti-inflammatory and immune modulation effects. In this study, we evaluated the anti-apoptotic effect of *C. lanceolata* on RPE cells by analyzing apoptosis-related factors, such as oxidative and carbonyl stress. *C. lanceolata* dose-dependently decreased the level of apoptosis in ARPE-19 cells induced by A2E and blue light co-treatment (Fig. 5B & Table 4). When comparing the anti-apoptotic potency between *C. lanceolata* and lutein (positive control drug), the normal cell population in 25 μM lutein treatment was 82.2 ± 0.95% and in 25 μg/mL *C. lanceolata* treatment was 83.5 ± 2.03%. As the comparison of 25 μM lutein and 25 μg/mL *C. lanceolata* treatment did not indicate a statistically significant difference, the anti-apoptotic potency of *C. lanceolata* was assumed to be similar to that of lutein. Based on its anti-apoptotic potency, we assessed the involvement of the Bcl-2 family-related intrinsic apoptosis pathway, the Keap1/Nrf2 anti-oxidative system, and carbonyl stress (Fig. 6). As shown in Fig. 5A, C*. lanceolata* effectively modulated the Bcl-2 family by increasing the levels of anti-apoptotic Bcl-2 proteins, such as Bcl-2 and Bcl-XL, while decreasing that of the pro-apoptotic protein, Bim. *C. lanceolata* not only stimulated the Keap1/Nrf2/HO-1 ARE pathway (Fig. 6B–D) but also inhibited the expression of 4-HNE (Fig. 6B).

## Conclusion

Based on these results, we obtained two findings. First, we found that the appropriate condition for an in vitro model of AMD is the treatment of ARPE-19 cells with 30 μM A2E and transillumination with 20 mW/cm^2^ blue light. This combination effectively induced oxidative and carbonyl stress, leading to the death of ARPE-19 cells. Second, we found that *C. lanceolata* shows promise as an anti-AMD agent. It effectively suppressed cellular stresses through Nrf2/HO-1 ARE activation and 4-HNE suppression, ultimately preventing apoptosis by deactivating the intrinsic apoptosis pathway.

### Supplementary Information

Supplementary Figure 1.

Supplementary Figure 2.

Supplementary Figure 3.

Supplementary Legends.

## Data Availability

All data and material in this manuscript will be made available on request to corresponding author.
