# Supplementary figures and images for: Oxidative and carbonyl stress induced AMD and Codonopsis lanceolata ameliorates AMD via controlling oxidative and carbonyl stress
Source: Sci Rep. 2024 Jul 15;14:16322. doi: 10.1038/s41598-024-67044-3 (PMC11251066; doi:10.1038/s41598-024-67044-3)

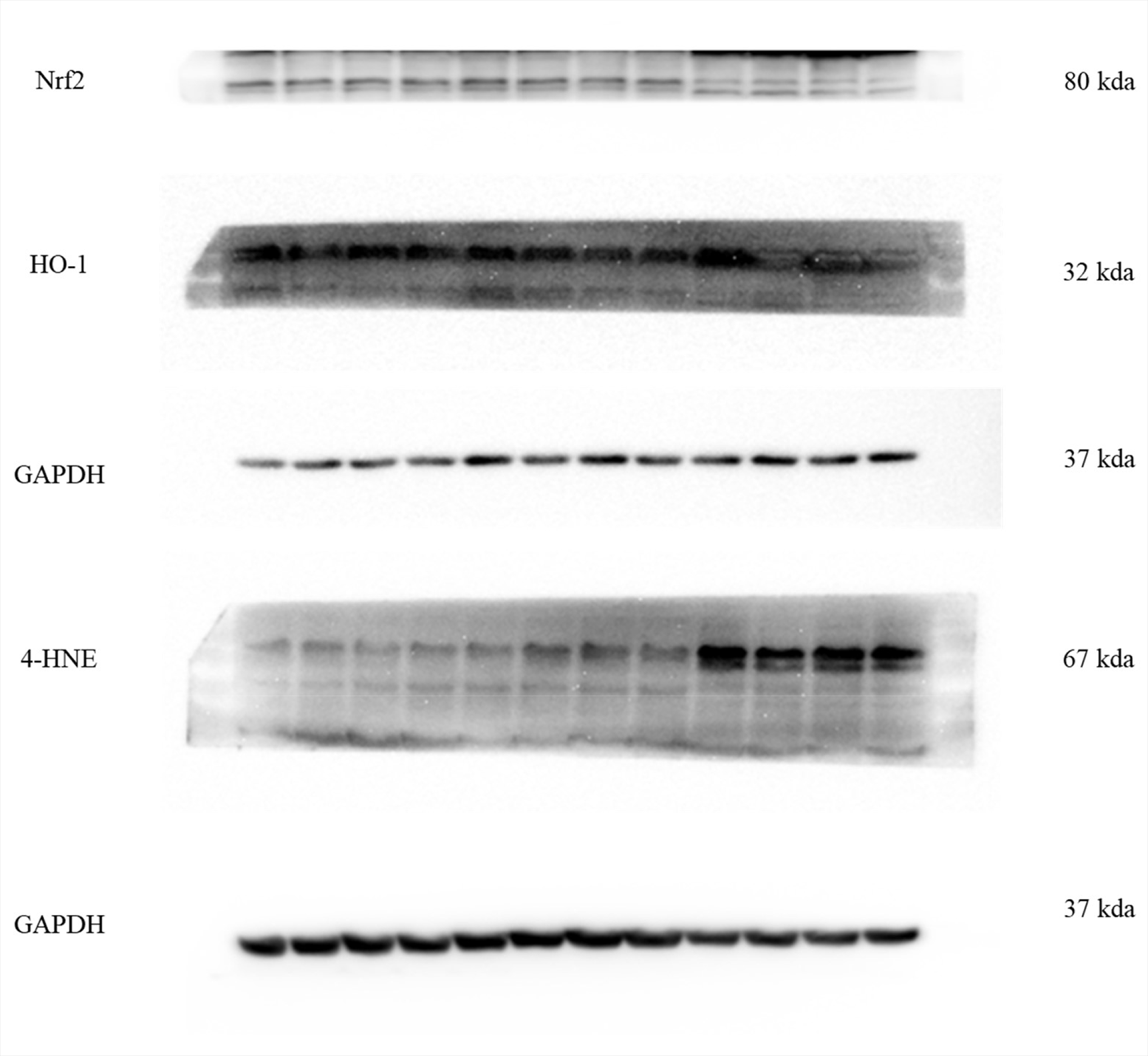

Supplement: Supplementary file 1 — Supplementary Figure 1. [file 41598_2024_67044_MOESM1_ESM.tif]

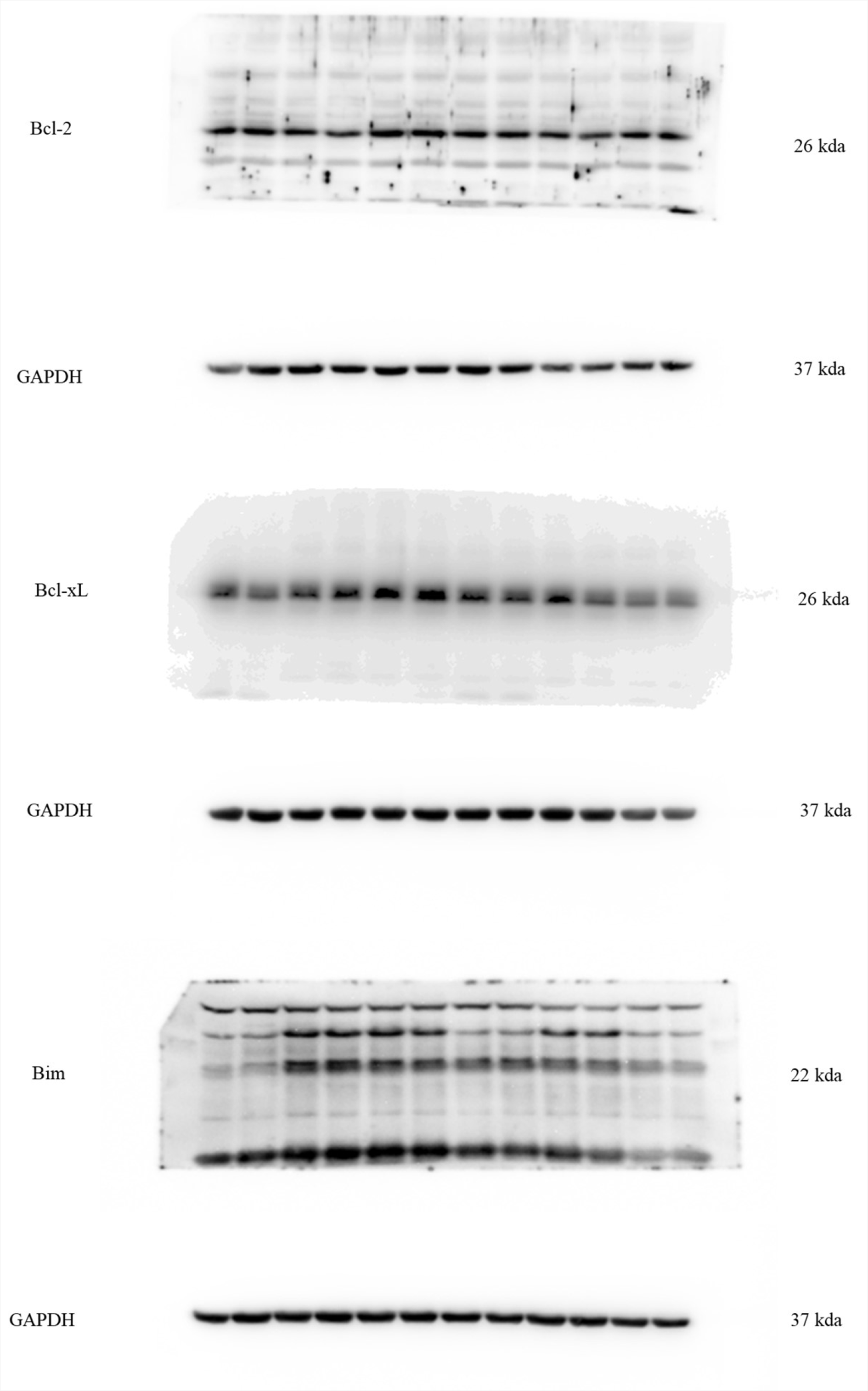

Supplement: Supplementary file 2 — Supplementary Figure 2. [file 41598_2024_67044_MOESM2_ESM.tif]

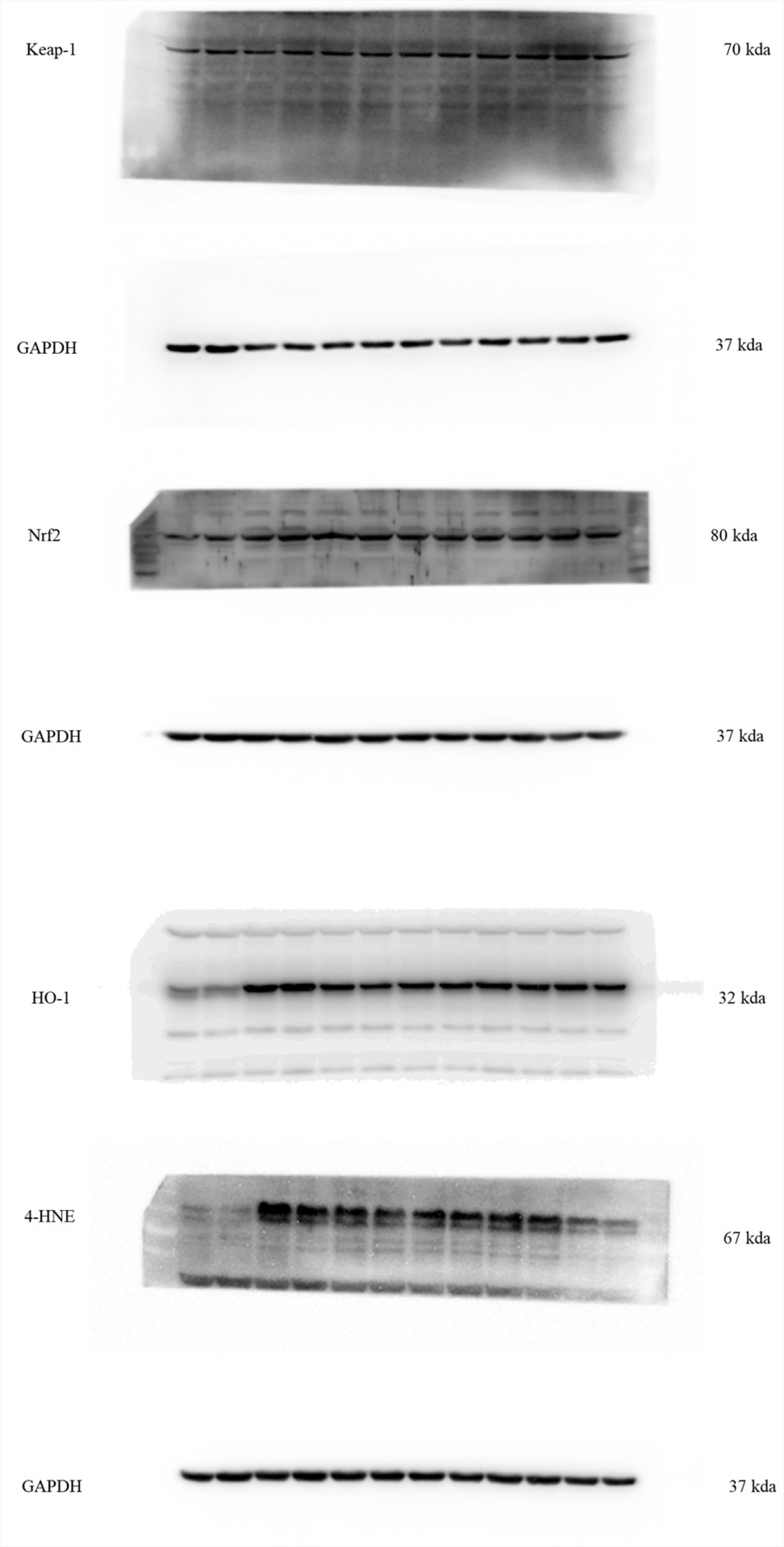

Supplement: Supplementary file 3 — Supplementary Figure 3. [file 41598_2024_67044_MOESM3_ESM.tif]
