# Supplementary material for: Oxidative and carbonyl stress induced AMD and Codonopsis lanceolata ameliorates AMD via controlling oxidative and carbonyl stress
Source: Sci Rep. 2024 Jul 15;14:16322. doi: 10.1038/s41598-024-67044-3 (PMC11251066; doi:10.1038/s41598-024-67044-3)
Supplement: Supplementary file 4 — Supplementary Legends. [file 41598_2024_67044_MOESM4_ESM.docx]

**Supplementary Figure S**1. The original data of western blots. The photo of western membrane of Figure 4.B. Western blotting was conducted two times for Nrf2/HO-1 and for 4-HNE. Before hybridization with primary antibodies one membrane was divided into two pieces for Nrf2 (80 kda) from 100 kda to 68 kda and for HO-1 (32 kda) from 48 kda to 20 kda and then images for two proteins such as Nrf2 and HO-1 were acquired. After that the membrane for HO-1 was stripped for aquation of GAPDH (37 kda) and then the image was acquired. Four-HNE (67 kda) and GAPDH (37 kda) were gotten from the other membrane.

**Supplementary Figure S2.** The original data of western blots. The photo of western membrane of Figure 6.A. Western blotting was conducted three times for each protein such as Bcl-2 (26 kda), Bcl-xL (26 kda), and Bim (22 kda). The image of GAPDH (37 kda) was acquired by the stripped each membrane.

**Supplementary Figure S3.** The original data of western blots. The photo of western membrane of Figure 6.B. Western blotting was conducted three times for Keap-1, Nrf2, and 4-HNE/HO-1. One membrane was divided into two pieces for Keap-1 (70 kda) from 135 kda to 50 kda and for GAPDH (37 kda) from 50 kda to 20 kda. Second membrane was divided into two pieces for Nrf2 (80 kda) from 135 kda to 45 kda. And the other one was divided into two pieces for 4-HNE (67 kda) from 100 kda to 50 kda and for HO-1 (32 kda) from 50 kda to 20 kda. After that the membrane for HO-1 was stripped for aquation of GAPDH (37 kda) and then the image was acquired.
